# Activation of Activin receptor-like kinases curbs mucosal inflammation and proliferation in chronic rhinosinusitis with nasal polyps

**DOI:** 10.1038/s41598-018-19955-1

**Published:** 2018-01-24

**Authors:** Lotta Tengroth, Julia Arebro, Olivia Larsson, Claus Bachert, Susanna Kumlien Georén, Lars-Olaf Cardell

**Affiliations:** 1 Divison of ENT Diseases, CLINTEC, Karolinska Institutet, Stockholm, Sweden; 2 Department of ENT Diseases, Karolinska University Hospital, Stockholm, Sweden; 3 Upper Airways Research Laboratory, Ghent University, Ghent, Belgium

## Abstract

Chronic rhinosinusitis with nasal polyps (CRSwNP) is a widespread disease causing obstruction of the nasal cavity. Its cause remains unclear. The transforming growth-factor beta (TGF-β) superfamily and their receptors, termed Activin receptor-like kinases (ALKs), have recently been suggested to play a role in local airway inflammation, but have so far not been evaluated in human nasal epithelial cells (HNECs) from CRSwNP patients. We demonstrated that ALK1–7 were expressed in the nasal polyp epithelium, and the expression of ALK1-6 was markedly elevated in polyps compared to nasal mucosa from healthy controls. Stimulation with the ALK ligand TGF-β1 decreased Ki67 expression in HNECs from CRSwNP patients, not evident in controls. Likewise, TGF-β1, Activin A and Activin B, all ALK ligands, decreased IL-8 release and Activin A and Activin B reduced ICAM1 expression on HNECs from CRSwNP patients, not seen in controls. Pre-stimulation with TGF-β1, Activin A, BMP4 and Activin B attenuated a TNF-α-induced ICAM1 upregulation on HNECs of CRSwNP. No effect was evident in controls. In conclusion, an increased expression of ALK1-6 was found on polyp epithelial cells and ligand stimulation appeared to reduce proliferation and local inflammation in polyps.

## Introduction

Chronic rhinosinusitis (CRS) is an inflammatory disease that affects more than 10% of the Western adult population^1^. According to the EPOS criteria, CRS can be divided into disease with or without nasal polyps (CRSwNP or CRSsNP)^2^. CRSwNP is an inflammatory disorder of the mucosa with unknown etiology. It is characterised by eosinophilic inflammation, Th2 cytokines, presence of pseudocyst formations and hyperplasia of the epithelium^2–4^. Multiple predisposing factors have been suggested to contribute to the polyp development in CRSwNP, but so far, the exact precipitating factors as well as the mechanisms involved remains unknown.

**Figure 1:**
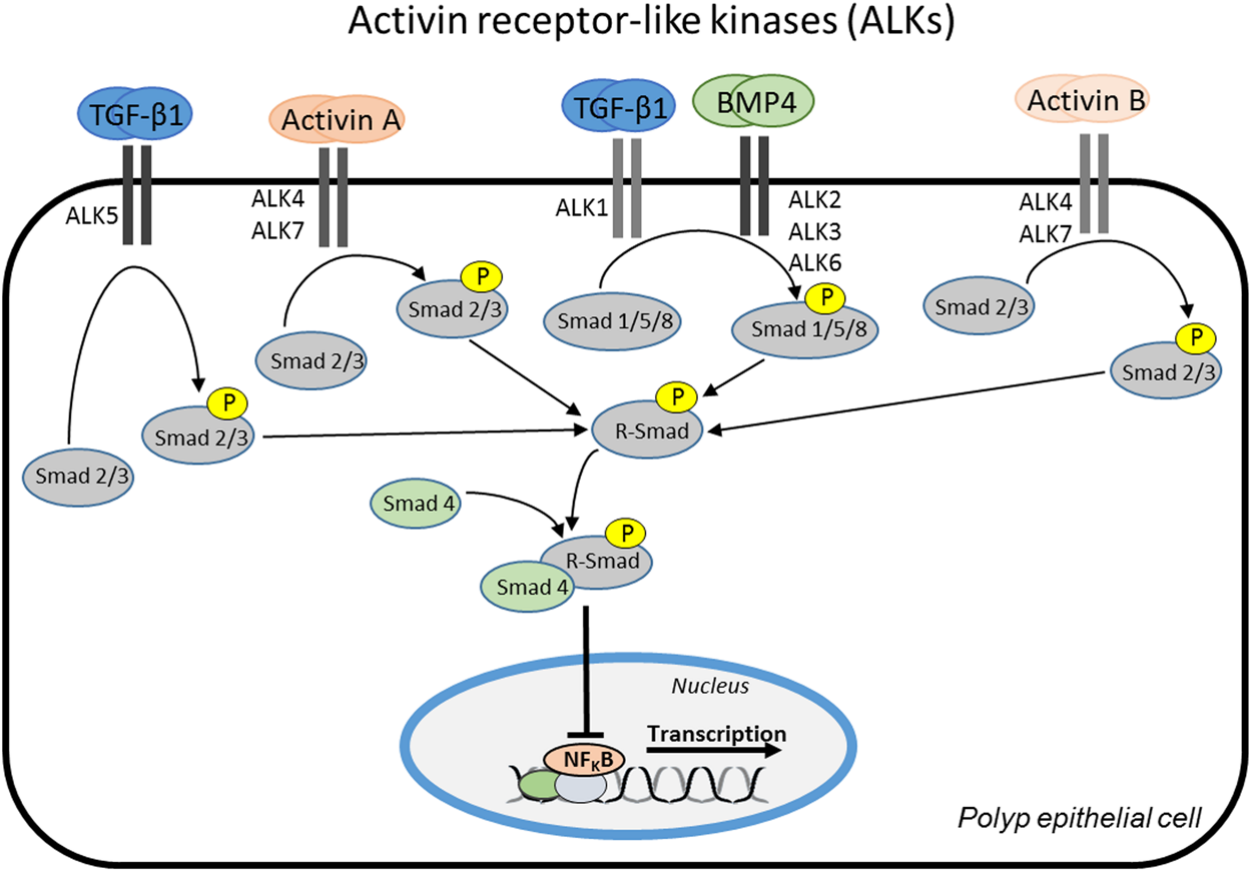
Schematic presentation of TGF-β-superfamily pathway in epithelial cells of polyps, including ligands, type I receptors and Smads. Formation of the ligand-receptor complex leads to phosphorylation of Smad. This activation blocks NF-κB and gene transcription. P indicates phosphorylation.

Recent investigations on upper airway inflammation have revealed a role for the transforming growth-factor beta (TGF-β) superfamily^5,6^. The members of this family can be divided in three subgroups: first the TGF-βs, second the activins/inhibins and third, the bone morphogenetic proteins (BMPs). The TGF-β ligands act through type I transmembrane serine/threonine kinase receptors, also termed Activin receptor-like kinases (ALKs), and type II transmembrane serine/threonine kinase receptors^7^. Seven ALKs, ALK1 to 7, have been identified in mammals to date^8^. Ligands can bind multiple ALKs but the affinities vary greatly. TGF-β1 binds ALK1 and 5 with high affinity. Activin A binds ALK2 and 4 with high affinity and ALK7 with moderate affinity^9^. BMP4 binds ALK3 and 6 with high affinity and ALK2 with moderate affinity. Finally, Activin B mainly aims for ALK4 and 7^10–12^. Ligands and receptors together form a heteromeric complex that phosphorylates and activates a number of intracellular Smads^7,13^ (Fig. 1). The outcome of activation is generally considered to be anti-inflammatory and profibrogenic^14^, but the knowledge of TGF-β-ALK complexes in CRSwNP is limited. TGF-β release is well studied and has been demonstrated to be expressed mainly in patients with CRSsNP. Low levels of TGF-β have been detected in patients with CRSwNP. At the same time, these patients with CRSwNP are known to have increased mucosal inflammation^15,16^. Activin A, another important member of the TGF-ß superfamily, show similar release patterns as TGF-β with low release in CRSwNP and higher release in CRSsNP^17^.

The presented study was designed to map the presence of ALKs in polyps of CRSwNP patients. Second, it aimed to characterise the outcome of potential receptor activation focusing on proliferation and mucosal inflammation, with an intention to evaluate the role of ALKs in polyp development.

## Results

### ALK expression in nasal mucosa

**Figure 2:**
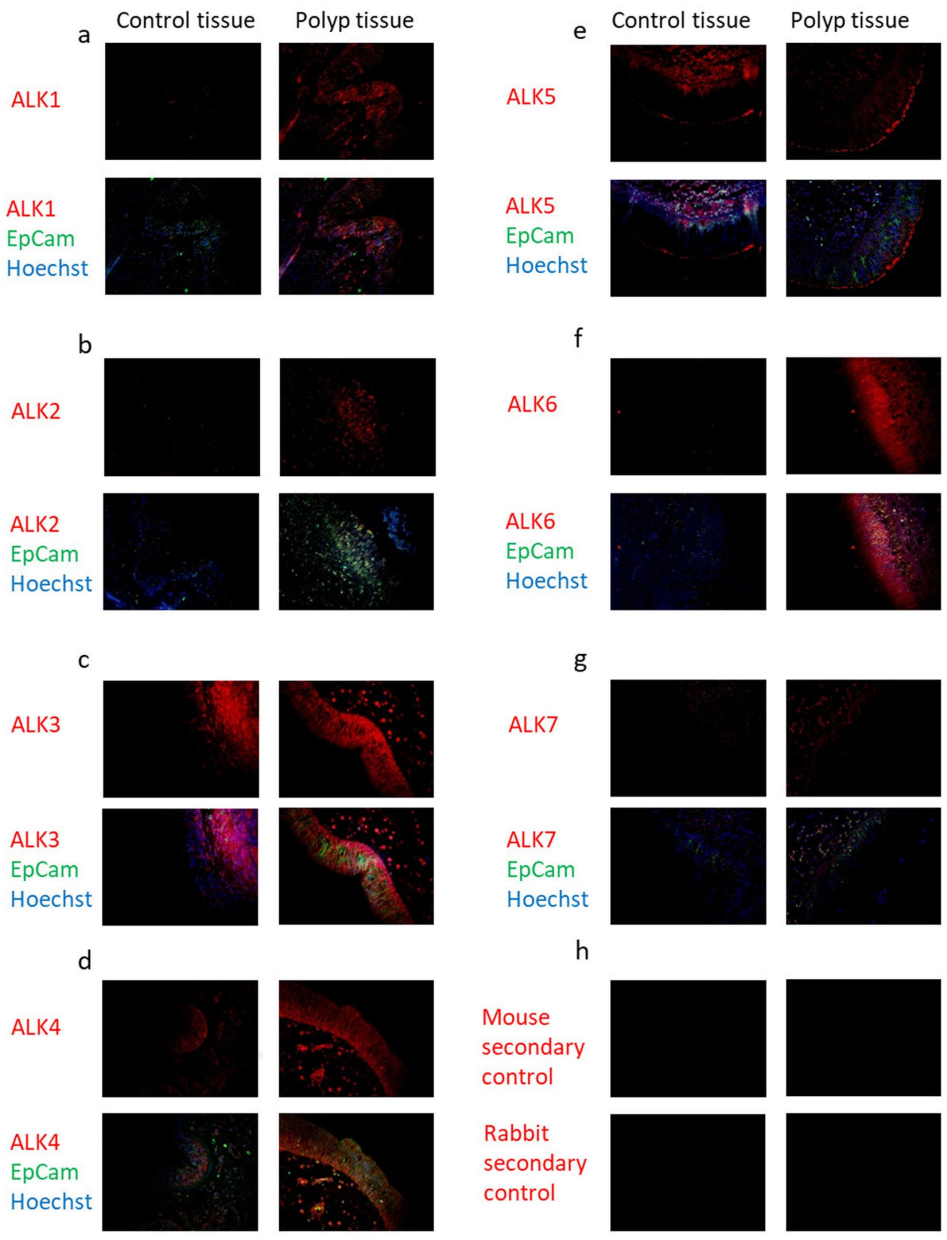
ALK expression in human nasal biopsies from patients with CRSwNP and healthy controls. ALK1 (**a**), ALK2 (**b**), ALK3 (**c**), ALK4 (**d**), ALK5 (**e**), ALK6 (**f**), ALK7 (**g**) Negative control (**h**). (n = 3–4). ALK (red), EpCam (green), nucleus/Hoechst (blue) and ALK localized in the nucleus (purple).

The presence of ALKs in nasal mucosa was verified by immunohistochemistry of nasal biopsies from patients with CRSwNP and healthy controls. ALK1 was moderately expressed in the mucosal layer of polyp tissues (Fig. 2a). ALK2 expression was seen in the epithelial layer in polyp tissues (Fig. 2b). A strong expression of ALK3 was seen in the epithelium and submucosal compartment of polyp tissue (Fig. 2c). ALK4 expression was mainly detected in the basal cells of the mucosa (Fig. 2d). Polyp tissue expressed ALK5 most abundantly in cells of the surface epithelium (Fig. 2e). ALK6 was expressed in the mucosal layer (Fig. 2f). Expression of ALK7 in polyp tissue was most abundant in basal cells of the epithelium and in the submucosal compartment (Fig. 2g). The expression of all receptors was only moderate (ALK3, ALK5) or low (ALK1-2, ALK4 and ALK7) in healthy control tissues (Fig. 2a–g). No unspecific binding could be detected (Fig. 2h).

**Figure 3:**
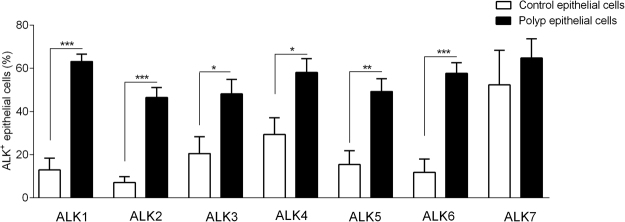
ALK expression. ALK expression on epithelial cells from patients with CRSwNP (n = 13) and healthy controls (n = 8) was determined using flow cytometry. Values: mean ± SEM. An unpaired *t-*test was used to compare the two sets of data. *p < 0.05, **p < 0.01, ***p < 0.001.

Since the expression of ALKs on HNECs is unidentified, we analysed the receptor expression on epithelial cells using flow cytometry for quantification. ALK1-6 expression was found to be significantly elevated on epithelial cells from polyp tissue, compared to corresponding expression from control tissue (Fig. 3). The expression of ALK7 was evident on epithelial cells from both tissues (Fig. 3).

### ALK ligands regulates abnormal proliferation on polyp epithelial cells

**Figure 4:**
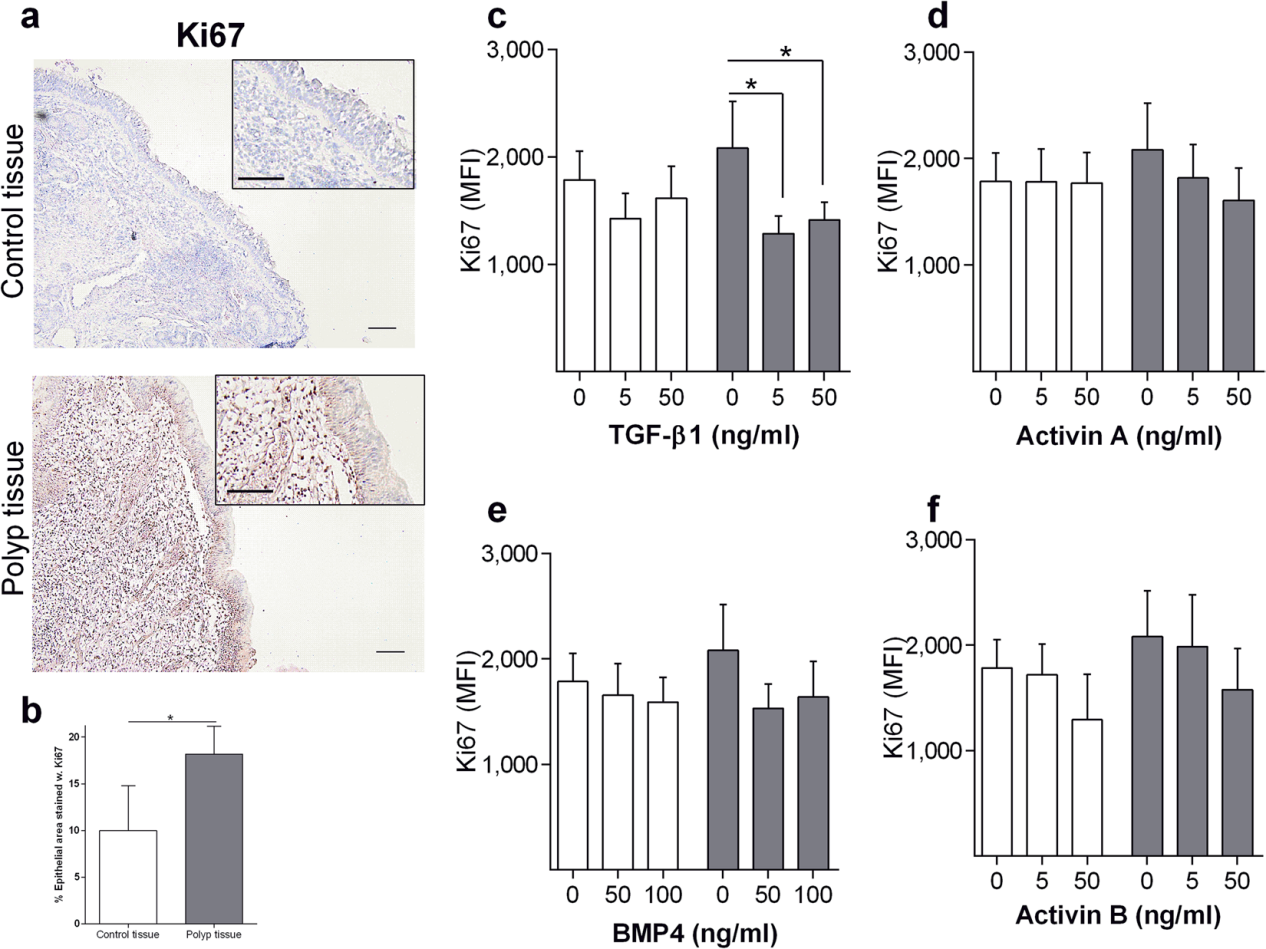
Regulation of Ki67 through ALK stimulation. Ki67 expression in nasal biopsies from patients with CRSwNP and healthy controls (n = 7) (**a**). Immunohistochemical staining and quantification of Ki67 (n = 7) (**b**). Ki67 expression on cultured epithelial cells stimulated with TGF-β1 (**c**), Activin A (**d**), BMP4 (**e**) or Activin B (**f**) for 24 h (n = 5). White bars (Control epithelial cells), gray bars (Polyp epithelial cells). Values: mean ± SEM. Friedman-test with Dunn’s post-test, was used for comparisons of more than two data sets. An unpaired *t-*test was used to compare two sets of data.

We continued evaluating the effects of ALK-stimulation. A schematic figure illustrates binding of ligands to ALKs, resulting in intracellular phosphorylation of Smads (Fig. 1). We started studying the potential of ALKs regulating abnormal epithelial cell proliferation. ALK expression was first evaluated on cultured epithelial cells demonstrating no change in ALK expression after *in vitro* culture over four passages (data not shown). This validated the method with nasal brushing and cell culturing for use in our forthcoming experiments. Ki67 immunostaining was confined to the basal cells of the polyp epithelium (Fig. 4a). Interestingly, Ki67 immunostaining was increased in polyp epithelium compared to control epithelium (Fig. 4b). Cultured HNECs were stimulated with multiple ALK ligands. Stimulation with TGF-β1 (binding to ALK1, 5) resulted in a significant downregulation of Ki67 expression on polyp epithelial cells (Fig. 4c). Stimulation with Activin A (ALK2, 4, 7), BMP4 (ALK2, 3, 6) and Activin B (ALK4, 7) did not downregulate Ki67 expression on polyp epithelial cells (Fig. 4d–f). Control epithelial cells showed no change in Ki67 upon stimulation (Fig. 4c–f).

### ALK ligands decrease IL-8 release from polyp epithelial cells

**Figure 5:**
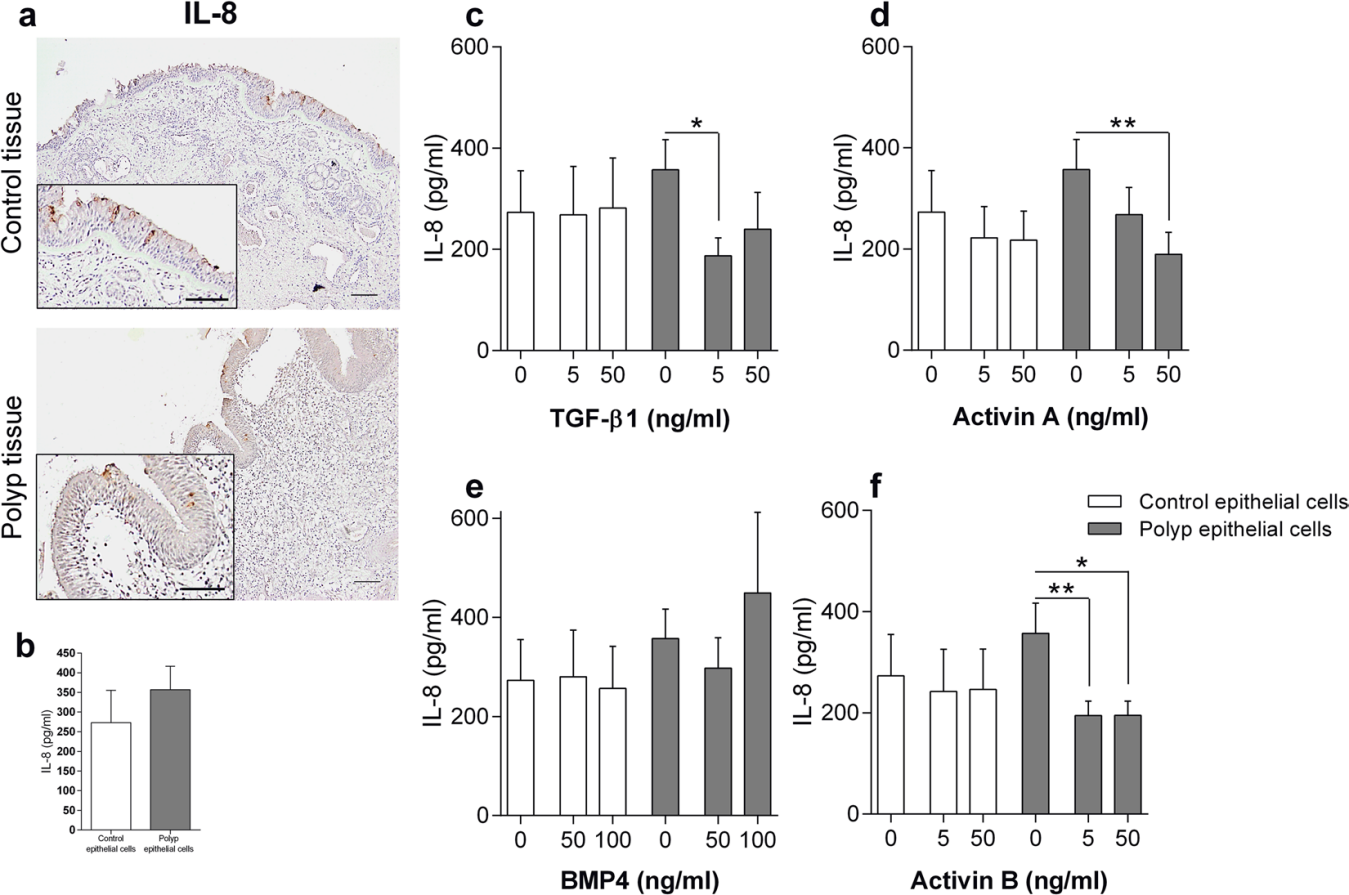
IL-8 release upon ALK stimulation. IL-8 expression in nasal biopsies from patients with CRSwNP and healthy controls (n = 8) (**a**). IL-8 release from cultured epithelial cells (**b**) or stimulated with TGF-β1 (**c**), Activin A (**d**), BMP4 (**e**) or Activin B (**f**) for 24 h (n = 5–6). Values: mean ± SEM. Friedman-test with Dunn’s post-test, was used for comparisons of more than two data sets. An unpaired *t-*test was used to compare two sets of data.

IL-8 expression was verified in polyp and control epithelium (Fig. 5a). IL-8 release was slightly elevated from polyp epithelial cells as compared to control (Fig. 5b). To study the ALK receptors effect on inflammation, HNECs were stimulated with TGF-β1, Activin A and B, respectively. This stimulation resulted in a significant reduction of IL-8 release from polyp epithelial cells, measured in supernatants (Fig. 5c, d and f). No reduction was observed from control epithelial cells (Fig. 5c, d and f). BMP4 stimulations did not result in any change in IL-8 release (Fig. 5e).

### TGF-β1, Activin A and B downregulate inflammation and inhibits TNF-α induced inflammation on polyp epithelial cells

**Figure 6:**
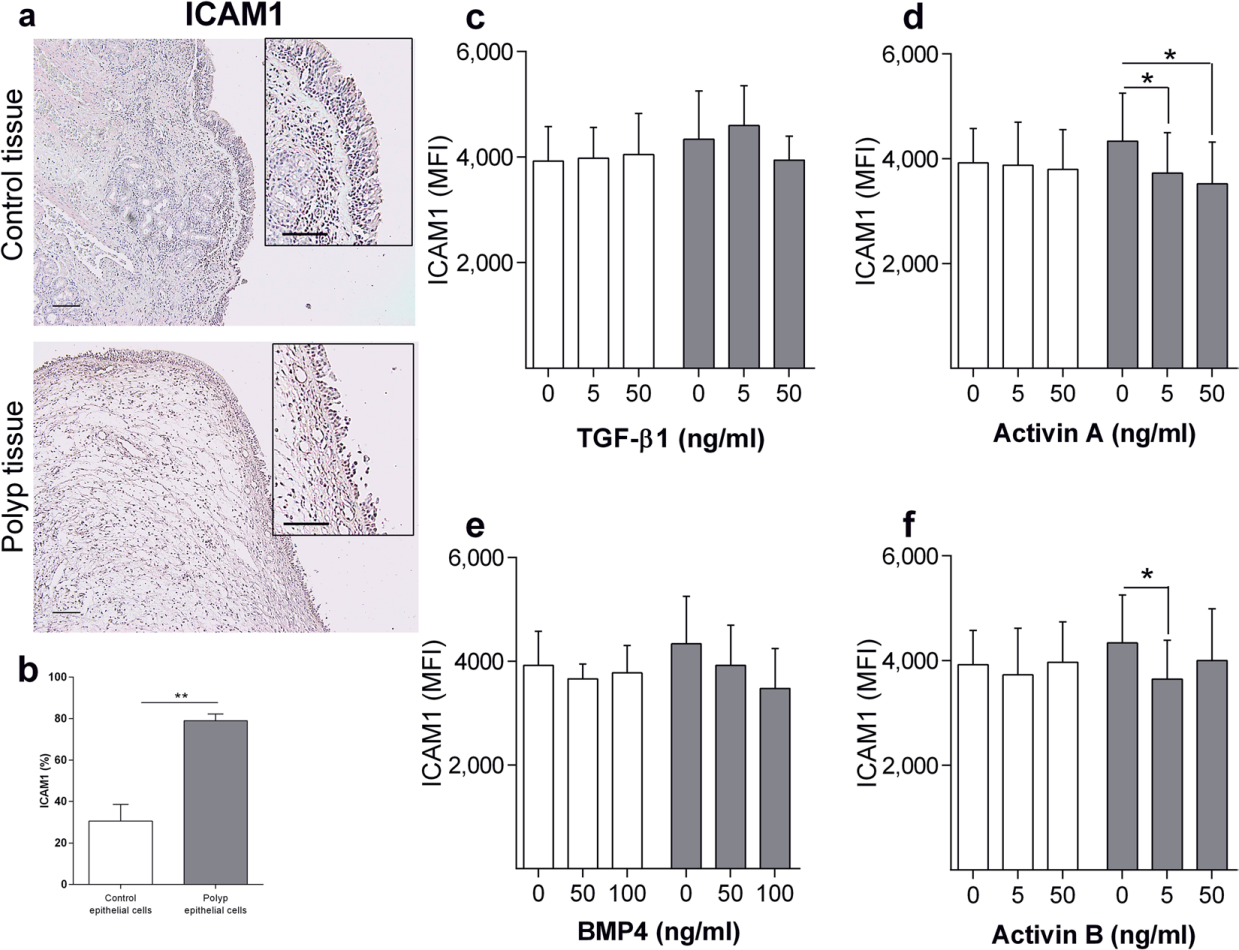
Regulation of ICAM1 through ALK stimulation. ICAM1 expression in nasal biopsies from patients with CRSwNP and healthy controls (n = 6–7) (**a**). ICAM1 expression on cultured epithelial cells (n = 5) (**b**) or stimulated with TGF-β1 (**c**), Activin A (**d**), BMP4 (**e**) or Activin B (**f**) for 24 h (n = 3). White bars (Control epithelial cells), gray bars (Polyp epithelial cells). Values: mean ± SEM. Friedman-test with Dunn’s post-test, was used for comparisons of more than two data sets. An unpaired *t-*test was used to compare two sets of data.

We continued evaluating the potential of ALKs regarding epithelial cell inflammation by studying ICAM1 expression on polyp and control tissue. ICAM1 expression was first verified in both polyp and control epithelium (Fig. 6a). Interestingly, ICAM1 expression was upregulated on polyp epithelial cells as compared to control (Fig. 6b). Activin A and B stimulation resulted in a downregulated ICAM1 expression on polyp epithelial cells (Fig. 6d and f). Control epithelial cells showed no change in ICAM1 expression upon ligand stimulation (Fig. 6c–f).

**Figure 7:**
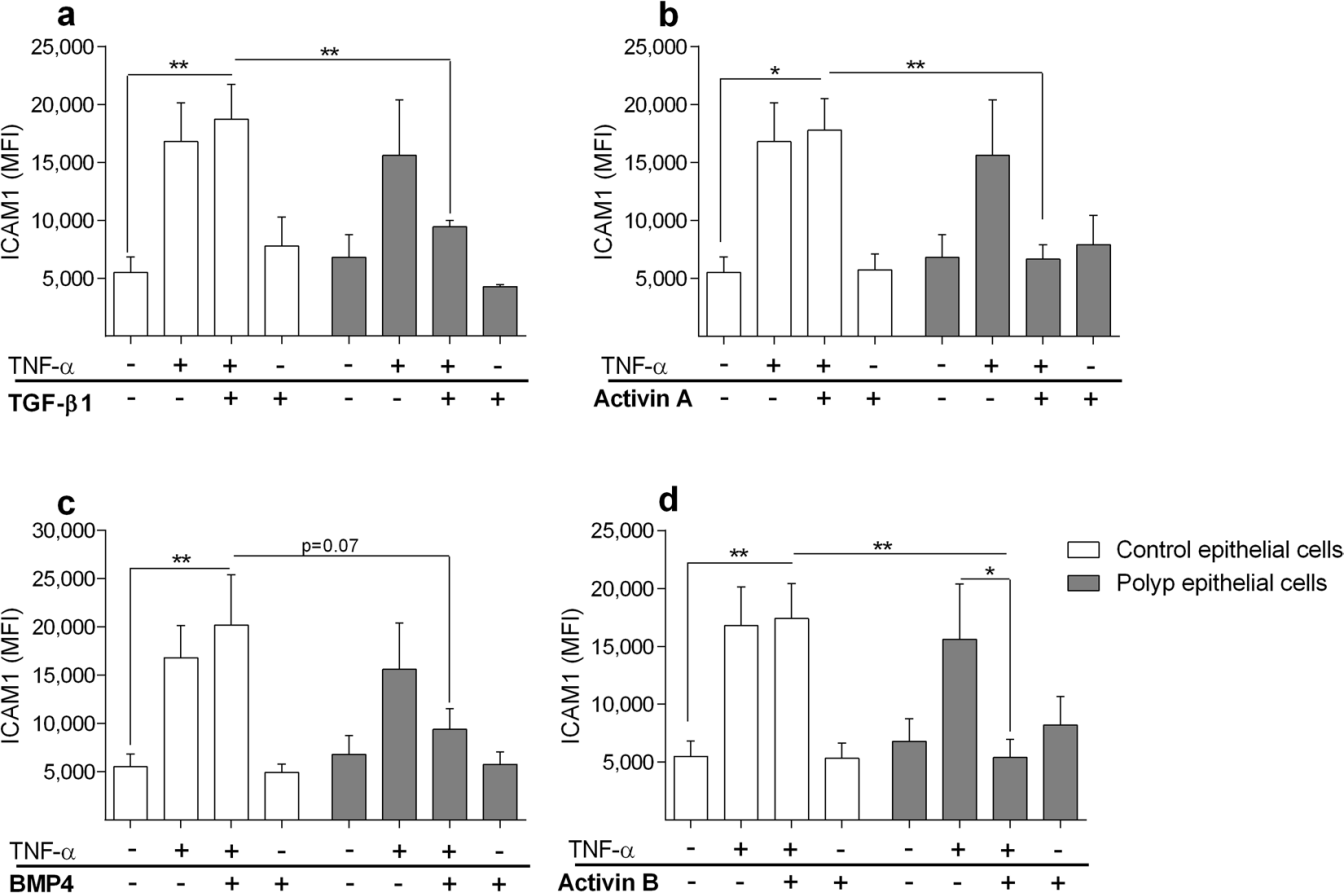
Effects of ALK stimulation on TNF-α induced ICAM1 expression. Cultured epithelial cells were stimulated with TGF-β1 (**a**), Activin A (**b**), BMP4 (**c**) or Activin B (**d**), for 20 h after which TNF-α was added for 4 h and ICAM1 expression was analysed (n = 5–7). Values: mean ± SEM. Friedman-test with Dunn’s post-test, was used for comparisons of more than two data sets. An unpaired *t-*test was used to compare two sets of data.

TNF-α, produced upon bacterial, fungal or viral infections, is known to cause more severe inflammation in patients with CRSwNP^18,19^. Thus, we tested the possibility that ALK ligands could inhibit this inflammatory response of epithelial cells. Exposure to TNF-α caused an upregulation of ICAM1 expression compared to unstimulated in both control and polyp epithelial cells (p = 0.002 for Control, p = 0.038 for Polyp). Pre-treatment for 20 h with TGF-β1, Activin A or Activin B, respectively, significantly inhibited TNF-α-induced ICAM1 expression on polyp epithelial cells compared to controls (Fig. 7a,b and d). Further, pre-treatment with BMP4 downregulated TNF-α-induced ICAM1 expression, although this did not reach statistical significance (Fig. 7c). In contrast, TNF-α-induced ICAM1 expression was increased on control epithelial cells after pre-treatment with TGF-β1, Activin A, BMP4 and Activin B (Fig. 7a–d).

## Discussion

The present study demonstrates the expression of a complete repertoire of ALK1-7 in the polyp epithelium. ALK1-6 were all upregulated on epithelial cells from polyps, compared to healthy controls. Stimulation of ALKs with their corresponding ligands resulted in a downregulation of the proliferation marker Ki67 and the inflammation markers IL-8 and ICAM1 on polyp epithelial cells. When stimulating polyp epithelial cells with TNF-α, mimicking infection, ICAM1 was upregulated. This upregulation could however be inhibited upon ALK-stimulation. This suggests that ALK activation might have anti-inflammatory effects on epithelial cells of nasal polyps.

The TGF-β superfamily has a well-established role in tissue development and pathogenesis. Together with cytokines produced in the tissue micromilieu, they have important roles in disease remodelling and inflammation^20,21^. ALK1 signalling and Smad phosphorylation was previously believed to be restricted to endothelial cells^22^. The present study demonstrates, for the first time, that polyp epithelial cells express high levels of 6 out of 7 ALKs and that the expression of ALK5 is most evident on the epithelium of polyp tissue. Previous studies have demonstrated Smad phosphorylation upon TGF-β and BMP stimulations, in epithelial cells of patients with CRSwNP. This emphasises that ALK signalling could affect gene expression and further molecular mechanisms in patients with CRSwNP. However, previous findings demonstrate low expression of ALK ligand TGF-β1 in polyps from CRSwNP compared to normal tissue^15,23^. In addition, polyps demonstrate low levels of Activin A whereas patients with CRSsNP demonstrate high levels of both TGF-β1 and Activin A. These studies indicate that ALK activation, through TGF-β1 and Activin A, is low in nasal polyps and possibly contributing to disease progression.

Several studies have demonstrated a correlation between Ki67 expression and severity of epithelial remodelling^24–26^. Our study demonstrated an upregulated expression of Ki67 in polyp epithelium and a downregulation of Ki67 expression after TGF-β1 stimulation, to levels expressed in control epithelial cells. A positive correlation between high Ki67 expression and polyp eosinophilia was recently discovered^27^. These results indicate that TGF-β1 could inhibit polyp epithelial cells with abnormal or defected proliferation, preventing cells to form a proper epithelial barrier. This effect could possibly be even more important in patients with high eosinophilia, a group considered more difficult to treat and require repeated surgery^28^.

Numerous studies have established that eosinophilic inflammation is predominantly involved in the pathogenesis of nasal polyps, especially in Western countries^29^. IL-8 release and ICAM1 expression on polyp epithelial cells is involved in the recruitment and transmigration of leucocytes, including eosinophils, to the polyp^30^. These markers were demonstrated to be elevated in the epithelial layer of polyps. Upon stimulation with Activin A, B or TGF-β1, the expression of IL-8 and ICAM1 was normalised to levels on control epithelial cells. Our results demonstrate a connection between stimulation with TGF-β1, Activin A and B and lowered inflammation in polyps. Interestingly, previous studies have shown a low release of both TGF-β1 and Activin A in polyps from patients with CRSwNP^23^, compared to controls. Activin A was specifically decreased at sites in the nasal polyps exhibiting a massive infiltration of B-cells^31^. Activin B has been shown to stimulate wound closure and cause reepithelialisation in mice via stimulation of the RhoA-JNK signalling pathway^32^. This implies that loss of TGF-β1, Activin A and B and their anti-inflammatory effects in polyps, could result in increased inflammation possibly driving further polyp development.

Patients with CRSwNP often experience worsening of the disease at certain times, and upper airway infections are often triggers of these periods. TNF-α is produced upon bacterial, fungal and/or viral infections and have been shown to cause more severe inflammation in patients with CRSwNP^18,19^. We here show that TNF-α increases inflammation by upregulating ICAM1 expression. Pre-stimulation with TGF-β1, Activin A and B reduced this TNF-α-induced ICAM1 upregulation on polyp epithelial cells. It is known that NF-κB plays a key role in ICAM1 and IL-8 gene transcription^33,34^. TGF-β and Activin A have previously been demonstrated to inhibit ICAM1 and IL-8 expression by blocking the activation of NF-κB^35,36^. We therefore hypothesise a role for TGF-β1, Activin A and B as protector against severe inflammation and the possible worsening of disease, upon infections in the polyps.

In summary, the present study demonstrates an increased expression of ALK1-6 in polyps compared to healthy turbinate tissues. Ligand stimulation appeared to reduce abnormal proliferation as well as local inflammation. Although our study demonstrated an anti-inflammatory function of these receptors it is interesting to note that a low release of both TGF-β1 and Activin have been demonstrated in patients with CRSwNP^15^. Further, virus-induced downregulation of TGF-β have previously been described^37,38^, possibly explaining the low TGF-β levels seen in patients with CRSwNP. The expression of both TGF-β and Activin A are upregulated in patients with CRSsNP compared to CRSwNP^17,39^. Hence, it is tempting to speculate that low expression of ALK ligands, like TGF-β1 or Activin A/B, contributes to loss of a controlled inflammation and epithelial proliferation, thereby perhaps driving polyp development. A further exploration of the role of ALKs in CRSwNP might open for novel therapeutic strategies in targeting the disease.

## Methods

### Subjects

The patients with CRSwNP were defined by endoscopic criteria and CT changes according to the European Position Paper on Rhinosinusitis and Nasal Polyps^2^. In all patients, steroids were withheld during at least 4 weeks (topically) and 8 weeks (systemically) prior participation. Patients characteristics are demonstrated in Table 1.

**Table 1:** Patients characteristics.

	Healthy Controls	Patients with CRSwNP
Sample size (no.)	20	26
Mean age (y)	33	49
Sex (M/F)	11/9	18/8
Asthma (no.)	0	6
Allergy (no.)	1	3
Smoker (no.)	0	2
Inhaled steroid (no.)	0	0

Nasal biopsies and/or epithelial cells derived from nasal brushing from 26 patients and 20 healthy controls were used. The epithelial cells derived from nasal brushing were analysed immediately with flow cytometry or cultured for stimulation experiments *in vitro*.

### Collection and immunohistochemistry of nasal biopsies

Nasal biopsies from polyp tissue and inferior or middle turbinate were obtained during functional endoscopic sinus surgery (FESS) or in local anaesthesia from patients with CRSwNP. Nasal biopsies from the inferior turbinate of healthy subjects were obtained in local anaesthesia, as previously described^40^. Biopsies were post-fixed in 4% PFA embedded in paraffin and subsequently cut into 5 μm sections using a microtome. The sections were deparaffinised in ultraclear, rehydrated in decreasing concentrations of ethanol and incubated in 0.1% Sudan black B (Sigma-Aldrich, St. Louis, USA) in 70% ethanol. Following heat-mediated antigen retrieval in citrate buffer, sections were permeabilised with 1% Triton X-100 (Sigma-Aldrich). For DAB staining, inhibition of the endogenous peroxidase activity was accomplished by treating the tissues with 1% H_2_O_2_ for 10 min; this was followed by incubation with 10% normal goat serum and 1% bovine serum albumin (BSA, Sigma-Aldrich) to block non-specific binding. Sections were either incubated with primary mouse anti-human antibodies against IL-8 (1:1000, ab18672), rabbit anti-human antibodies against Ki67 (1:5000, ab15580) or ICAM1 (1:100, ab109361, all from Abcam) overnight and subsequently incubated for 1 h with a secondary goat anti-mouse antibody (1:200, Vector Laboratories, Peterborough, United Kingdom) or goat anti-rabbit antibody (1:200, Vector Laboratories). Sections were incubated with Avidin-Biotin-Complex (Vectastain, PK 6100, Vector Laboratories) followed by 3,3′-diaminobenzidine (DAB) (SK-4100, Vector Laboratories), according to the manufacturer’s instructions. The slides were counterstained with Mayer’s Haematoxylin. Images were captured with a Nikon Digital Sight DS-U1 camera, coupled to a Nikon Eclipse TE2000-U microscope, using NIS Elements F. 2.20 software. Image analyse was carried out using ImageJ. Images of immunohistochemical stains of Ki67, ICAM and IL-8 were initially deconvoluted to separate the DAB and haematoxylin channels. The threshold of the DAB channel was set to a constant value for all pictures, and the total epithelial area with staining was measured.

For immunofluorescence, 3% BSA was used to block non-specific binding. Mouse anti-human antibodies against ALK1 (1:25, ab51870), ALK2 (1:25, ab78414), ALK4 (1:25, ab78415), ALK6 (1:50, ab78417), or rabbit anti-human antibodies against ALK3 (1:50, ab38560), ALK5 (1:100, ab31013), ALK7 (1:100, ab111121), were added and incubated for 1 h at room temperature and subsequently incubated with a secondary AlexaFluor 555-conjugated donkey anti-mouse antibody (1:200, ab150110) or secondary AlexaFluor 555-conjugated donkey anti-rabbit antibody (1:200, ab150066 all from Abcam, Cambridge, UK). Nucleus was visualised using Hoechst staining (33258, Sigma). The epithelial layer of the nasal mucosa was located using EpCam (Supplement Fig. 1). As a negative control, mouse or rabbit antibody diluent was used. Imaging was performed using an Olympus Provis microscope, connected to an Olympus U-PS camera.

### Isolation and culture of human nasal epithelial cells

Human nasal epithelial cells (HNECs) from polyp and turbinate tissue from patients with CRSwNP, as well as from healthy controls, were isolated by nasal brushing as previously described^41^. Cells were analysed immediately with flow cytometry or maintained in collagen-coated tissue culture flasks in Keratinocyte Serum-Free Medium (KSFM) (Gibco, Paisley, UK), supplemented with 0.005 μg/mL epidermal growth factor (Gibco), 0.05 mg/mL bovine pituitary extract (Gibco), 20 U/mL penicillin, 20 μg/mL streptomycin and 0.25 μg/mL Fungizone (complete KSFM). All cells were cultured in a humidified chamber at 37 °C with 5% CO_2_. Cells from passages 2–6 were used, and were all positive for EpCam, an epithelial-specific adhesion molecule.

### Cell stimulation

HNECs were seeded on 24-well culture plates (200,000 cells/well) in 1 mL complete KSFM and incubated overnight. A pilot dose-response study with agonists included was first performed. Cells were subsequently cultured for 24 h with two different concentrations of TGF-β1, Activin A, BMP4 or Activin B (R&D Systems, Abingdon, United Kingdom) or cultured for 20 h with TGF-β1, Activin A, BMP4 or Activin B, after which TNF-α (R&D Systems) was added. Cell-free supernatants were analysed for cytokine release. Cell were analysed for Ki67 and ICAM1 expression using flow cytometry.

### Flow cytometry

The cells from nasal brushing, not used for culture, were washed and centrifuged, after which the supernatant was aspirated and discarded. The Vybrant Apoptosis Assay Kit #3 from Molecular Probes (Eugene, OR) was used to assess viable cells (>90%). The epithelial cells were then gated based on their EpCam expression, revealing ~50% epithelial cells.

The following mouse monoclonal antibodies were used for staining: unlabelled ALK1 (clone MM0015-8G33, Abcam), unlabelled ALK4 (clone 544310, R&D Systems), ALK6-PE (clone 477914, R&D Systems), ALK7-APC (clone 810506, R&D Systems), ICAM1-FITC (11C81, R&D Systems) and Ki67-PE-Cy7 (clone B56, BD, San Jose, USA), rabbit polyclonal unlabelled ALK5 (Abcam) and goat polyclonal unlabelled ALK2 (R&D Systems) and ALK3-APC (R&D Systems). Unlabelled antibodies were used for staining together with goat anti-mouse-FITC, donkey anti-goat-FITC and polyclonal goat-PE. Isotype controls relevant for each antibody were used for background staining. Transcription factor buffer set (BD) was used to detect the intranuclear proteins, according to instructions of the manufacturer. For both extracellular and intranuclear staining, cells were incubated with antibodies or isotype controls for 20 min at room temperature, thereafter washed and resuspended in phosphate buffered saline (PBS) (Gibco) with 1% formaldehyde. Epithelial cells were gated based on forward and side scatter properties and analysed on an LRSFortessa™ analyser (BD). Events in the range of 20,000–50,000 were collected. Data were analysed with FlowJo Analysis Software (©Tree Star, Inc, Ashland, USA).

### ELISA

The levels of IL-8 in supernatants of stimulated HNECs were measured in duplicates using a DuoSet ELISA kit (R&D Systems) according to the manufacturer’s instructions. The minimum detectable IL-8 concentration for the kit was 15.6 pg/ml.

### Statistical analysis

Data was analysed using GraphPad Prism Software (San Diego, CA, USA). Results are expressed as mean ± SEM. n equals the number of subjects. Data were analysed using Friedman test for non-parametric data followed by a Dunn’s multiple comparison post-test (for comparisons of more than two data sets). An unpaired *t-*test was used to compare two sets of data. A p-value of 0.05 or less was considered statistically significant. *p < 0.05, **p < 0.01, ***p < 0.001.

### Ethics statement

The study was approved by the Ethics Committees of Karolinska Institutet, Stockholm, Sweden. All participants gave their written informed consent, while all procedures were conducted according to the principles expressed in the Declaration of Helsinki.

## Electronic supplementary material

Supplementary Dataset 1
