# Supplementary material for: Activation of Activin receptor-like kinases curbs mucosal inflammation and proliferation in chronic rhinosinusitis with nasal polyps
Source: Sci Rep. 2018 Jan 24;8:1561. doi: 10.1038/s41598-018-19955-1 (PMC5784055; doi:10.1038/s41598-018-19955-1)
Supplement: Supplementary file 1 — Supplementary Dataset 1 [file 41598_2018_19955_MOESM1_ESM.pdf]

Activation of Activin receptor-like kinases curbs mucosal inflammation and proliferation in chronic rhinosinusitis with nasal polyps

Authors: Lotta Tengroth[1], Julia Arebro[1,2], Olivia Larsson[1], Claus Bachert[1,2,3], Susanna Kumlien Georén[1], Lars-Olaf Cardell[1,2]*

Affiliations:

[1]Divison of ENT Diseases, CLINTEC, Karolinska Institutet, Stockholm, Sweden

[2]Department of ENT Diseases, Karolinska University Hospital, Stockholm, Sweden

[3]Upper Airways Research Laboratory, Ghent University, Ghent, Belgium

*Correspondence to: Lars-Olaf Cardell, Division of ENT Diseases, Department CLINTEC, Karolinska Institutet, Karolinska University Hospital, 141 86 Stockholm, Sweden, phone: +46 70 770 99 26; fax: +46 8 774 79 07; e-mail: lars-olaf.cardell@ki.se

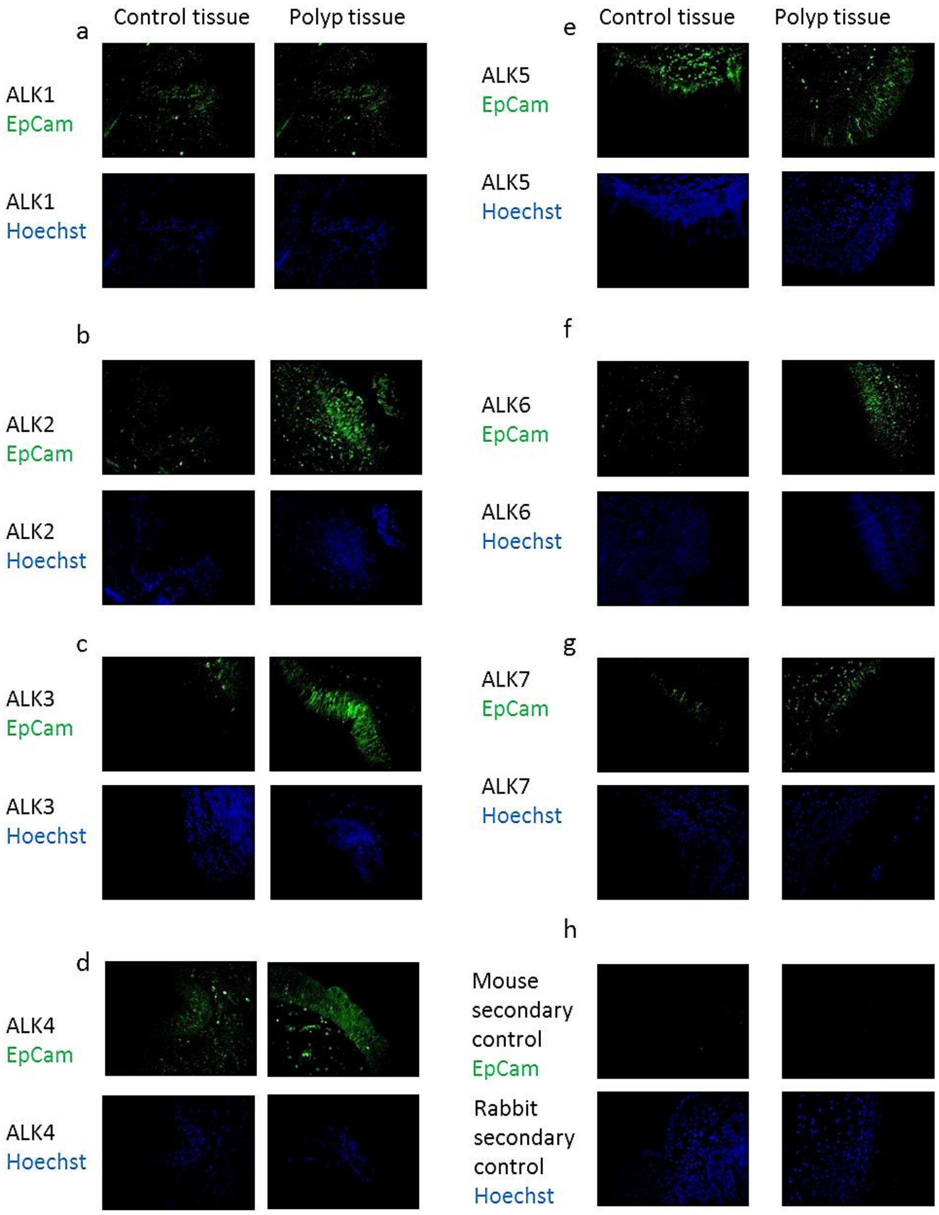

Supplement Figure 1. **The epithelial layer of the human nasal biopsies from patients with CRSwNP and healthy controls.** ALK1 (**A**), ALK2 (**B**), ALK3 (**C**), ALK4 (**D**), ALK5 (**E**), ALK6 (**F**), ALK7 (**G**) Negative control (**H**). (n = 3-4). EpCam (green) and nucleus using Hoechst staining (blue).
